# Spatiotemporal Changes and Influencing Factors of Hand, Foot, and Mouth Disease in Guangzhou, China, From 2013 to 2022: Retrospective Analysis

**DOI:** 10.2196/58821

**Published:** 2024-08-02

**Authors:** Jiaojiao Liu, Hui Wang, Siyi Zhong, Xiao Zhang, Qilin Wu, Haipeng Luo, Lei Luo, Zhoubin Zhang

**Affiliations:** 1 School of Public Health, Southern Medical University, Guangzhou, China; 2 Department of Communicable Disease Control and Prevention, Guangzhou Center for Disease Control and Prevention, Guangzhou, China; 3 School of Public Health, Sun Yat-sen University, Guangzhou, China

**Keywords:** hand, foot, and mouth disease, spatial analysis, space-time scan statistics, geographically and temporally weighted regression, infectious disease

## Abstract

**Background:**

In the past 10 years, the number of hand, foot, and mouth disease (HFMD) cases reported in Guangzhou, China, has averaged about 60,000 per year. It is necessary to conduct an in-depth analysis to understand the epidemiological pattern and related influencing factors of HFMD in this region.

**Objective:**

This study aims to describe the epidemiological characteristics and spatiotemporal distribution of HFMD cases in Guangzhou from 2013 to 2022 and explore the relationship between sociodemographic factors and HFMD incidence.

**Methods:**

The data of HFMD cases in Guangzhou come from the Infectious Disease Information Management System of the Guangzhou Center for Disease Control and Prevention. Spatial analysis and space-time scan statistics were used to visualize the spatiotemporal distribution of HFMD cases. Multifactor ordinary minimum regression model, geographically weighted regression, and geographically and temporally weighted regression were used to analyze the influencing factors, including population, economy, education, and medical care.

**Results:**

From 2013 to 2022, a total of 599,353 HFMD cases were reported in Guangzhou, with an average annual incidence rate of 403.62/100,000. Children aged 5 years and younger accounted for 93.64% (561,218/599,353) of all cases. HFMD cases showed obvious bimodal distribution characteristics, with the peak period from May to July and the secondary peak period from August to October. HFMDs in Guangzhou exhibited a spatial aggregation trend, with the central urban area showing a pattern of low-low aggregation and the peripheral urban area demonstrating high-high aggregation. High-risk areas showed a dynamic trend of shifting from the west to the east of peripheral urban areas, with coverage first increasing and then decreasing. The geographically and temporally weighted regression model results indicated that population density (*β*=−0.016) and average annual income of employees (*β*=−0.007) were protective factors for HFMD incidence, while the average number of students in each primary school (*β*=1.416) and kindergarten (*β*=0.412) was a risk factor.

**Conclusions:**

HFMD cases in Guangzhou were mainly infants and young children, and there were obvious differences in time and space. HFMD is highly prevalent in summer and autumn, and peripheral urban areas were identified as high-risk areas. Improving the economic level of peripheral urban areas and reducing the number of students in preschool education institutions are key strategies to controlling HFMD.

## Introduction

Hand, foot, and mouth disease (HFMD) is an infectious disease caused by enteroviruses, which mostly occurs in children younger than 5 years of age. The main characteristics are small herpes or small ulcers on the hands, feet, mouth, and other parts. Most children recover spontaneously in about a week, but a few children can face complications, such as myocarditis, pulmonary edema, and aseptic meningoencephalitis. In some severe cases, the illness progresses quickly, resulting in death [1]. Due to the scarcity of effective therapeutic medications, symptomatic care is mainly used. HFMD was first reported in New Zealand in 1957. In the past 30 years, it has shown the characteristics of widespread epidemics and infections in the Asia-Pacific region, causing more than 2 million hospitalizations in Asia every year as well as sporadic outbreaks in Europe and the United States [2-5]. Since May 2008, the disease has been included in the management of Category C notifiable infectious diseases under China’s “Infectious Disease Prevention and Control Law.” The average number of reported cases per year exceeds 1 million, ranking it among the top 3 notifiable infectious diseases in China all year round. Dynamic sequence analysis shows that the reported incidence of HFMD is increasing in China, with the largest increase occurring in the southern region and the southeast coastal region as a hot spot and high-risk area [6,7]. Guangzhou is a regional central city in South China and China’s southern gateway to the world. It has a dense population and frequent mobility and has a typical marine monsoon climate in the south subtropics. Many factors have jointly contributed to the prevalence of HFMD in Guangzhou. The number of HFMD incidences in Guangzhou is among the highest in China all year round [8,9].

Due to differences of socioeconomic factors in different regions, the spread and distribution of the HFMD virus show significant spatial heterogeneity [10]. In this case, spatial epidemiology plays a key role as an important branch of epidemiology, using geographic information system (GIS) technology to accurately describe and analyze the spatial distribution characteristics and development patterns of HFMD in different regions [11]. Then, we can more accurately determine key areas for epidemic prevention and control, guide the deployment and adjustment of prevention and control work, effectively control the spread of the epidemic, and protect public health to the greatest extent.

So far, the spatiotemporal distribution of HFMD and its influencing factors have been extensively studied [12-14], but few articles have combined the understanding of spatiotemporal distribution with the control of potential influencing factors. To control the epidemic of HFMD more effectively, more in-depth research in this area is necessary. Conventional linear regression methods are often used to examine the influence of potential factors, but they have some limitations; they cannot capture the changing trends of data that may exist in different regions or time points, and data between adjacent locations may interact with each other. On the contrary, geographically weighted regression (GWR) is an extension of ordinary least squares regression (OLS) for spatial data analysis. It can consider the impact of spatial distribution on the model, fit a local model for each observation point to realize the spatial change of the model, and reflect the impact of geographical location more accurately, thereby improving the reliability and interpretability of analysis results [15]. Moreover, the geographically and temporally weighted regression (GTWR) model builds upon GWR by incorporating a temporal dimension. This enhancement enables the model to account for both spatial and temporal nonstationarity, providing a comprehensive understanding of the spatiotemporal dynamics of HFMD and its influencing factors [16]. Therefore, this study described the spatiotemporal distribution of HFMD in Guangzhou through spatial autocorrelation analysis and space-time scan statistics analysis. Additionally, it analyzed the impact of population, economy, education, and medical care on the spatiotemporal distribution of HFMD in Guangzhou from 2013 to 2022 through multivariate OLS, GWR, and GTWR to provide a reference for precise prevention and control of HFMD in southern China.

## Methods

### Study Area

**Figure 1.**
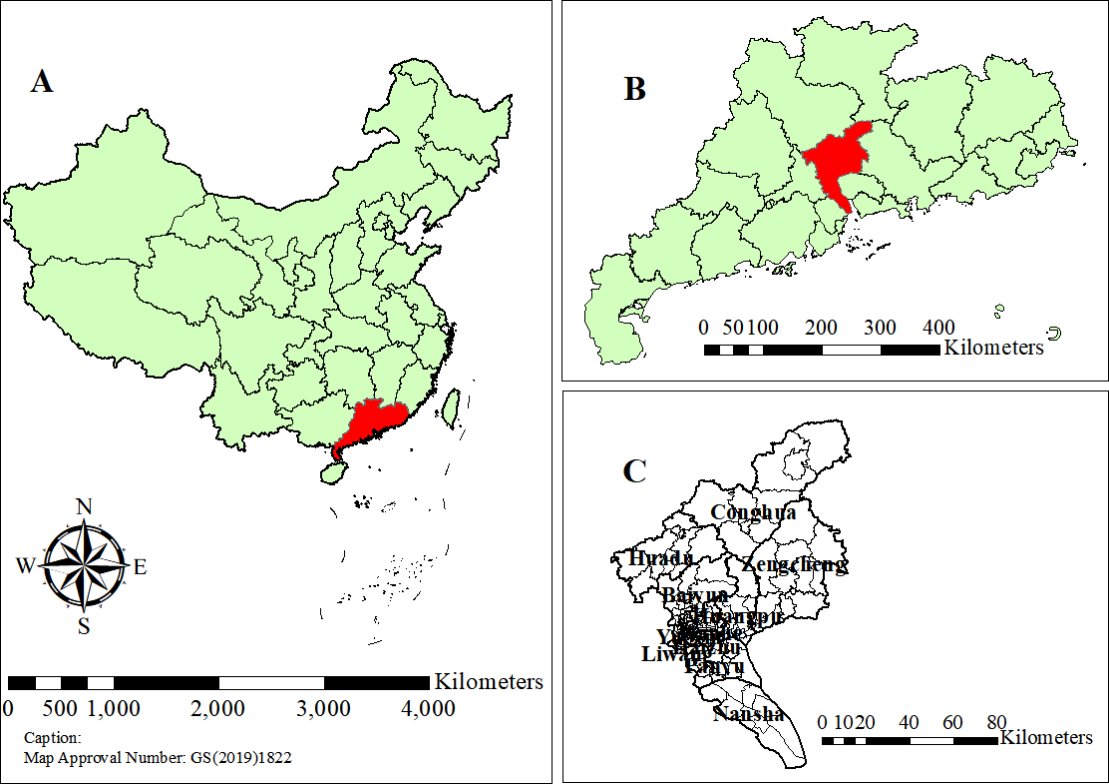
Location of the study area. The location of (A) Guangdong Province in China, (B) Guangzhou city in Guangdong Province, and (C) 11 county-level administrative districts in Guangzhou city.

Guangzhou is located in the lower reaches of the Pearl River in South China, bordering on the South China Sea (latitude 22°26′ to 23°56′N; longtitude 112°57′ to 114°3′E). Guangzhou has 11 county-level administrative districts, a total of 136 streets, and 35 towns (Figure 1). Due to its coastal geographical location as well as population diversity and mobility, Guangzhou has become a prosperous economic center, with a permanent population of more than 18.73 million in 2022. The annual per capita GDP exceeds 150,000 Yuan (USD 20,637). According to the Guangzhou Urban Master Plan (2017‐2035) [17], Guangzhou’s 11 districts are divided into 3 levels. The central urban area includes Liwan, Yuexiu, Tianhe, and Haizhu districts; the subcenter includes Baiyun, Huangpu, and Nansha districts; and the peripheral areas include Huadu, Panyu, Conghua, and Zengcheng districts.

### Data Sources

Since the Ministry of Health included HFMD in the management of notifiable infectious diseases in 2008, every HFMD case is required to be reported to the notifiable infectious disease information system in real time. Information on HFMD cases in Guangzhou from 2013 to 2022 was obtained through the Infectious Disease Information Management System of the Guangzhou Center for Disease Control and Prevention. Only HFMD cases whose current address was in Guangzhou were included in the study. The population, economy, education, and medical care data of each administrative districts came from the Guangzhou Municipal Bureau of Statistics [18].

### Spatial Autocorrelation Analysis

Spatial autocorrelation analysis is a statistical method used to explore the distribution of data in geographical space. It mainly covers the following two aspects: (1) global spatial autocorrelation analysis evaluates the overall degree of spatial aggregation or dispersion trend of a phenomenon in the entire region, and (2) local spatial autocorrelation analysis identifies and quantifies local clustering or dispersion patterns of a phenomenon in geographic space [19]. The global Moran’s *I* was used to measure the strength of the overall spatial autocorrelation. The values ranged from −1 to 1, with values close to 1 indicating positive spatial autocorrelation, values close to −1 indicating negative spatial autocorrelation, and values close to 0 indicating no spatial autocorrelation. The local Moran’s *I* (*I_i_*) was used to measure the correlation between a region and its neighboring regions. The values ranged from −1 to 1, with positive values indicating high-high (HH) or low-low aggregation areas and negative values indicating high-low or low-high aggregation areas. HH aggregation area was where an area had a higher incidence than the average incidence and was surrounded by adjacent areas higher than average incidence. The high-low aggregation area was clustered with an area with an incidence above the average incidence, but surrounded by adjacent areas with incidences below the average. After statistically testing the local indicator of spatial association (LISA) through the *Z* test, the LISA aggregation map was drawn to intuitively show the different aggregation forms in geographical space.

The global Moran’s *I* and *I_i_* were calculated as follows [20]:


$$I=\frac{n}{W}\frac{\sum_{i=1}^{n}\sum_{j=1}^{n}w_{ij}\left(x_{i}-\bar{x}\right)\left(x_{j}-\bar{x}\right)}{\sum_{i=1}^{n}{\left(x_{i}-\bar{x}\right)}^{2}}\;\;\;\;\;\;\;\;\;\left(1\right)$$

$$I_{i}=\frac{\left(x_{i}-\bar{x}\right)\sum_{j=1}^{n}w_{ij}\left(x_{j}-\bar{x}\right)}{\sum_{j=1}^{n}{\left(x_{j}-\bar{x}\right)}^{2}}\;\;\;\;\;\;\;\;\;\;\;\;\;\;\;\;\;\;\;\;\;\;\;\left(2\right)$$

where *n* was the number of geographic cells; *W* was the weight matrix; *w_ij_* was the spatial weight between geographic cells *i* and *j*; *x_i_* and *x_j_* were the values of the variables over geographic cells *i* and *j*; and *x̄* was the mean value of the variables.

### Space-Time Scan Statistics

Space-time scan statistics is a statistical method for detecting spatiotemporal aggregation. Its basic principle is to divide the study area into different spatiotemporal windows by establishing dynamically adjustable cylinder windows. The base of the cylinder represents the geographical space, and the height of the cylinder represents the time range [21]. Considering the epidemiological characteristics of HFMD [22], this study used the maximum radius not exceeding 30% of the total population as the spatial scale and the maximum aggregation of 3 months as the time scale. Contrast distributions were generated through stochastic simulation; the expected number of events per spatiotemporal window was calculated and compared with the number of observed events; thus, the test statistic log-likelihood ratio (LLR) was calculated. The *P* values were calculated by Monte Carlo simulation. When *P*<.05, a greater LLR value indicated a greater probability that the scanning window area was an aggregation area. The area with the largest LLR value was selected as a class I aggregation area, and other areas were selected as the class II aggregation areas.

### Influencing Factors Analysis

The incidence of HFMD is related to many social factors. Based on previous studies and combined with local characteristics [23-25], four aspects including population, economy, education, and medical care were selected to analyze the impact of the spatiotemporal distribution of HFMD cases. Specific indicators are available in Multimedia Appendix 1.

GWR and GTWR are spatial statistical methods used to explore the spatial relationship between dependent variables and independent variables. Considering that factors such as population, economy, education, and medical care are unevenly distributed in space, GWR takes geographical coordinates as part of the regression model. When fitting the model, it will adapt to local changes for each geographical location and fit different parameters accordingly.

GWR model and GTWR model were calculated as follows [26,27]:


$$y_{i}=\beta_{0}\left(u_{i},v_{i}\right)+\sum_{j}^{k}\beta_{j}\left(u_{i},v_{i}\right)x_{ij}+\varepsilon_{i}\;\;\;\;\;\;\;\;\;\;\;\left(3\right)$$

$$y_{i}^{t}=\beta_{0}^{t}\left(u_{i},v_{i}\right)+\sum_{j}^{k}\beta_{j}^{t}\left(u_{i},v_{i}\right)x_{ij}^{t}+\varepsilon_{i}^{t}\;\;\;\;\;\;\;\;\;\;\left(4\right)$$

where *y_i_* is the observed value of the dependent variable at the spatial location; *β*_0_(*u_i_, v_i_*) is the intercept term; *β_j_*(*u_i_*, *v_i_*) is the coefficient of the independent variable *j* at the spatial location *i*; *x_ij_* is the observed value of the independent variable *j* at the spatial location *i*; and *ε_i_* is the error term representing the unexplained part of the model and is the observed value of the dependent variable at time *t* and spatial position *i*; the other indicators are the same.

The corrected Akaike information criterion (AIC) and *R*^2^ values were used to evaluate the OLS, GWR, and GTWR models. A lower AIC value indicates that the model has a good fit to the data while considering complexity, and the closer the *R*² value is to 1, the stronger the ability of the model to explain the variables.

### Statistical Analysis

Multifactorial OLS and GWR analyses were performed using R (version 4.3.2; R Foundation for Statistical Computing). Spatial autocorrelation analysis was performed using ArcGIS (version 10.8; Environmental Systems Research Institute). GTWR analysis was performed using the GTWR plugin, developed by Professor Bo Huang's team. Space-time scanning analysis was performed using SaTScan 10.1 (developed by Martin Kulldorff together with Information Management Services Inc) [28].

### Ethics Approval

The study was approved by the ethics review committee of Guangzhou Centers for Disease Control and Prevention (GZCDC-ECHR-2023P0072). The incidence data used in this study were derived from the reported cases of HFND and had been anonymized and deidentified during the data export process.

## Results

### HFMD Prevalence

**Figure 2.**
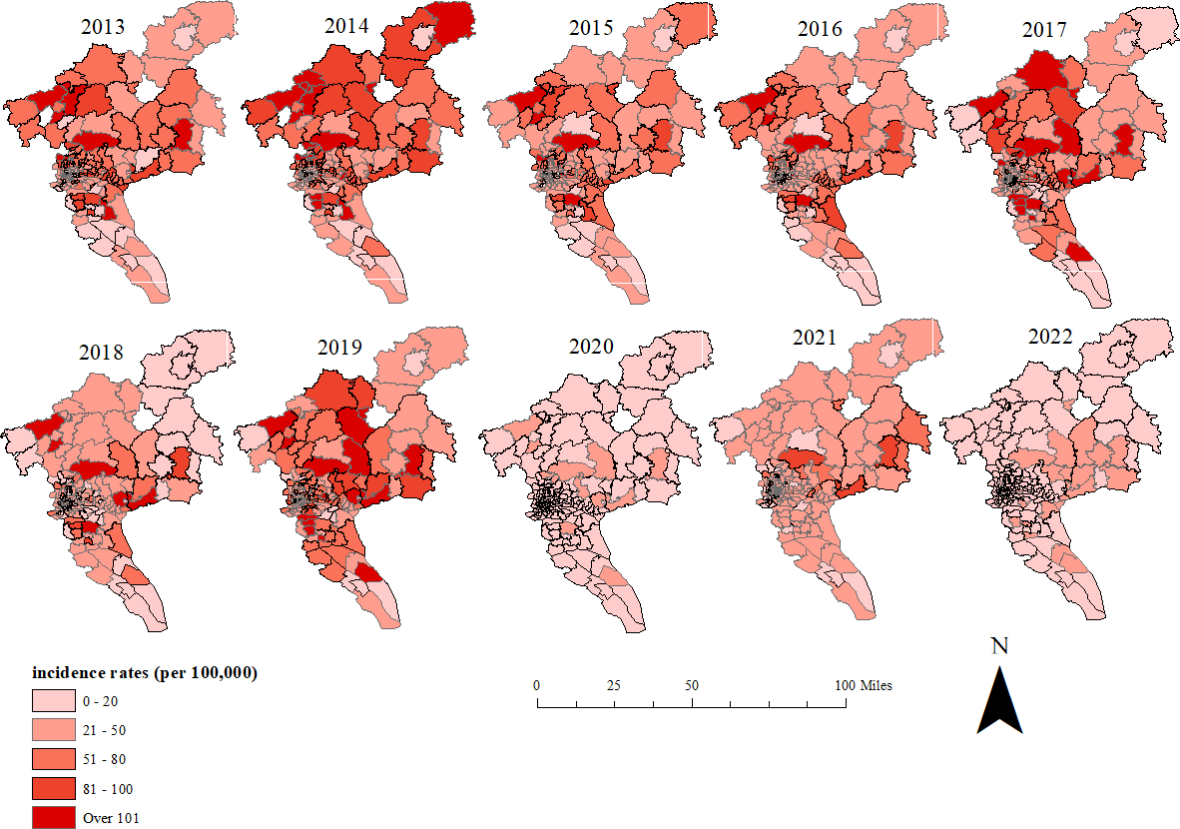
The prevalence and trend of hand, foot, and mouth disease (HFMD) in Guangzhou from 2013 to 2022.

**Figure 3.**
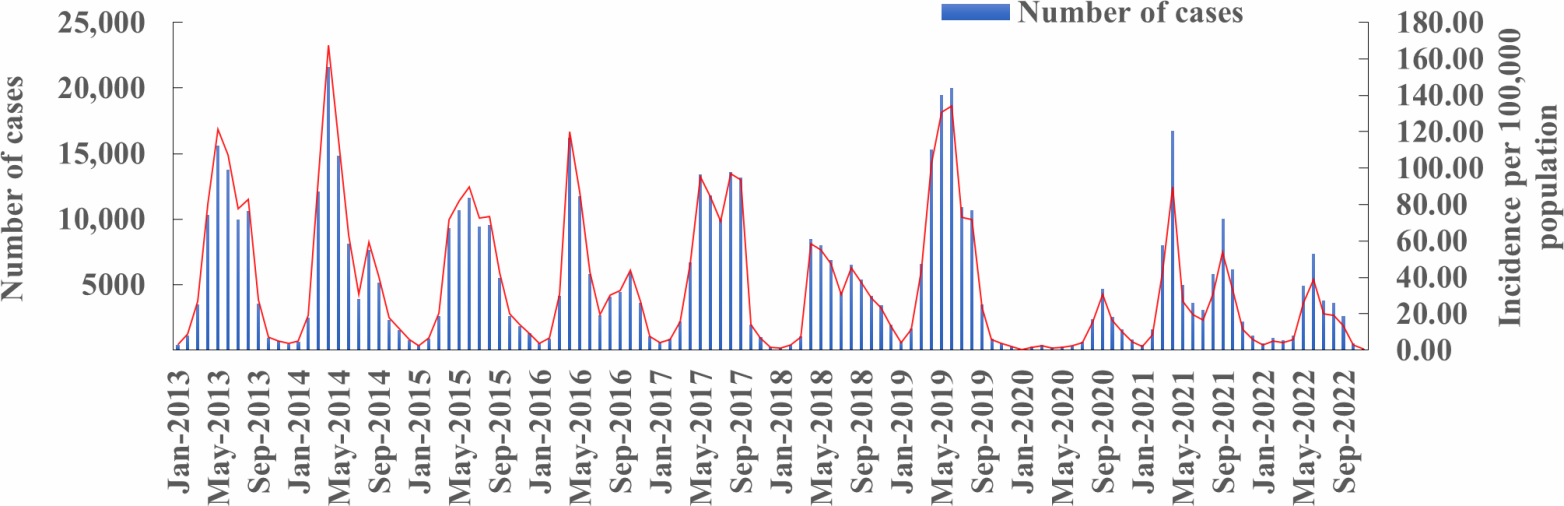
Reported hand, foot, and mouth disease (HFMD) incidence rates by street/township in Guangzhou from 2013 to 2022.

From 2013 to 2022, a total of 599,353 cases of HFMD were reported in Guangzhou, Guangdong Province. The ratio of male to female incidences was 1.51:1. The median age of patients was 2.42 (IQR 1.33‐1.75) years, and 93.64% (561,218/599,353) were children aged 5 years and younger (Multimedia Appendix 2). The ratio of scattered children (aged< 3 years) to nursery children (aged 3‐6 years) was 1.87:1. Cases aged ≥10 years accounted for only 1.55% (9304/599,353) of the total number of cases. The average annual reported incidence rate was 403.51/100,000, and the average annual change percentage was −12.94%, showing an overall downward trend. HFMD cases were characterized by a bimodal distribution, with the peak period from May to July and the second peak period from August to October (Figure 2). The average annual incidence of HFMD in Huadu District was the highest (614.90/100,000), which was 2.51 times higher than that in Yuexiu District (244.81/100,000). The top 3 distrcits with cumulative cases were Baiyun District (118,005 cases), Panyu District (79,379 cases), and Zengcheng District (68,637 cases). Multimedia Appendix 3 and Figure 3 show the changes of HFMD incidence at the district and street (town) levels.

### Spatial Autocorrelation Analysis of HFMD Cases

**Figure 4.**
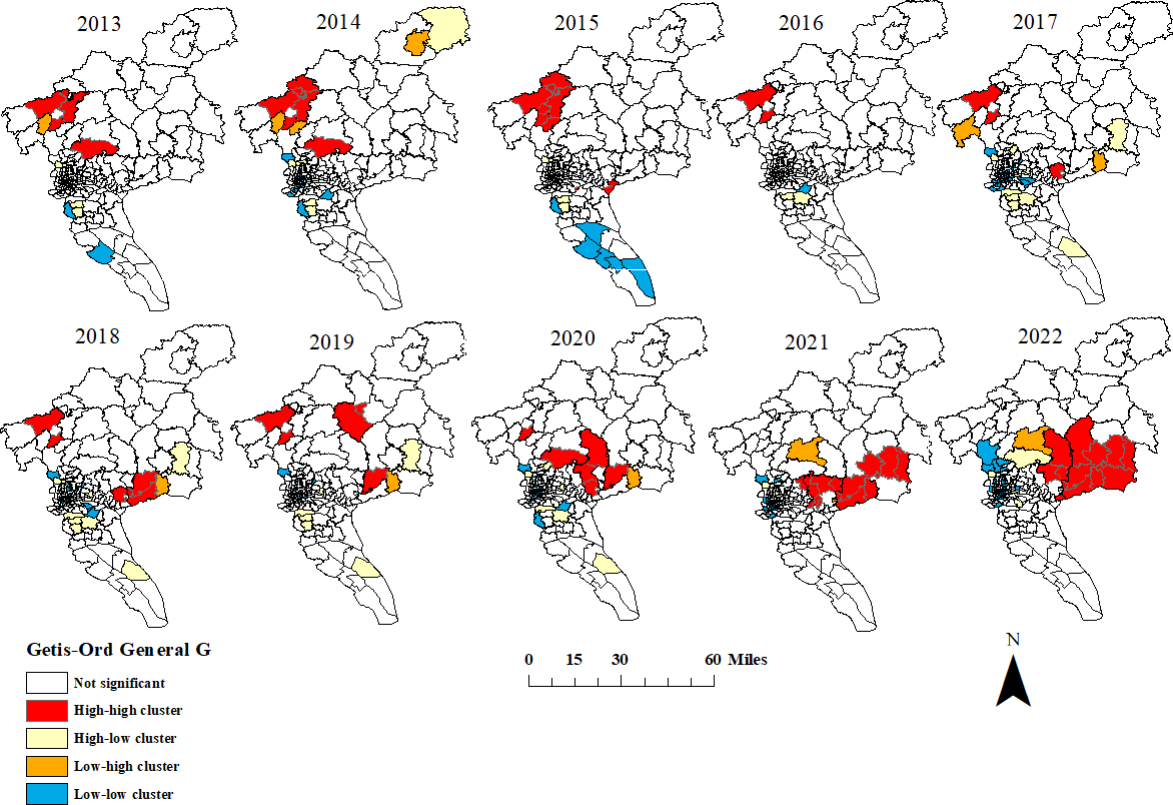
Local spatial association analysis of hand, foot, and mouth disease (HFMD) incidence in Guangzhou from 2013 to 2022.

Table 1 shows that the global Moran’s *I* ranges from 0.048 to 0.29, except for the Moran’s *I* of 0.015 (*P*=.45) in 2020, and the 10 years global Moran’s *I* value is 0.059 (*P*=.02), indicating that the HFMD cases in Guangzhou are not randomly distributed and have a high degree of positive spatial autocorrelation and a spatial aggregation trend. In Figure 3, the LISA aggregation map shows that the HH aggregation area centered on Shiling Town and Xinhua Street in the western region of Guangzhou continued to exist from 2013 to 2017, but the number of streets (towns) involved decreased. The HH aggregation area was centered on Yongning Street and Licheng Street in the eastern region from 2018 to 2022, and the number of streets (towns) involved has increased. From 2013 to 2016, the LL aggregation area was mainly distributed in the southern region, and from 2017 to 2022, it was mainly distributed in the southwest region of Guangzhou (Figure 4).

**Table 1.** The results of the spatial autocorrelation analysis of the hand, foot, and mouth disease (HFMD) incidence from 2013 to 2022.

Year	Moran’s *I*	Variance	Z test	*P* value	High-high	High-low	Low-high	Low-low
2013	0.073	0.00078	2.82	.005	4	5	1	2
2014	0.072	0.0008	2.746	.006	5	8	3	20
2015	0.103	0.00082	3.814	<.001	8	7	0	6
2016	0.151	0.00082	5.489	<.001	2	4	0	1
2017	0.056	0.00079	2.19	.03	3	11	2	32
2018	0.070	0.00074	2.793	.005	5	10	1	15
2019	0.048	0.00076	1.969	.049	5	6	1	10
2020	0.015	0.00079	0.754	.45	6	7	1	22
2021	0.151	0.00082	5.489	<.001	13	6	2	19
2022	0.295	0.00081	10.574	<.001	12	9	1	30
Average	0.059	0.00081	2.289	.02	3	11	0	27

### Space-Time Scanning Analysis of HFMD Cases

**Figure 5.**
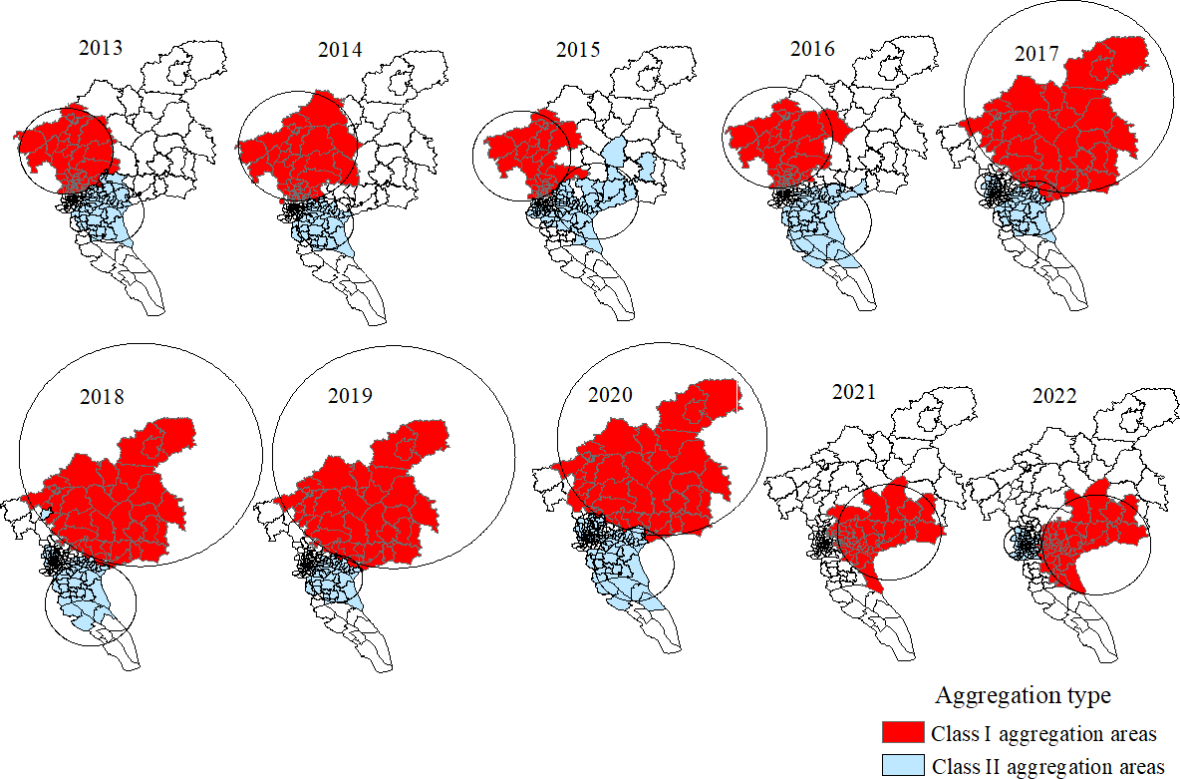
Spatiotemporal scan cluster analysis of hand, foot, and mouth disease (HFMD) in Guangzhou from 2013 to 2022.

As shown in Table 2 and Figure 5, space-time scanning analysis detected 1 to 3 cluster areas every year, with a total of 23 clusters, all of which were statistically significant (*P*<.001). Class I aggregation area involved an average of 41 streets and towns per year.

From a temporal perspective, clusters were observed in all months except from December to March, and class I clusters mainly occurred from May to July. From a spatial perspective, the centers of class I aggregation areas from 2013 to 2016 were all located in Huadu District, including Xinya Street, Huashan Town, Xiuquan Street, and Huacheng Street, with an average radiation radius of 27.74 km, covering Huadu District, Baiyun District, and some streets (towns) in Tianhe District. From 2017 to 2020, the class I aggregation area moved and expanded to the northeast of Guangzhou, with Lutian Town in Conghua District as the center, with an average radiation radius of 64.1 km, covering Conghua District, Zengcheng District, Huadu District, Baiyun District, and some streets (towns) in Huangpu District. From 2021 to 2022, the class I aggregation area moved and decreased to the southeast of Guangzhou, with Yongning Street and Xintang Town in Zengcheng District as the center, with an average radiation radius of 28.06 km, covering Zengcheng District, Huangpu District, Panyu District, and some streets (towns) in Tianhe District.

**Table 2.** Statistics of spatiotemporal scan cluster analysis of hand, foot, and mouth disease (HFMD) in Guangzhou from 2013 to 2022.

Year and gathering center		Aggregation radius (km)	Gathering time (months)	Number of streets/towns	RR^a^	LLR^b^	*P* value
**2013**							
	Xinya Street	24.62	May-July	42	4.03	9773	<.001
	Hualong Town	17.16	May-July	47	2.37	2949	<.001
**2014**							
	Huashan Town	31.38	April-June	41	4.45	13,266	<.001
	Xinzao Town	14.55	April-June	45	2.69	4498	<.001
**2015**							
	Xiuquan Street	25.91	May-July	31	3.37	5281	<.001
	Yunpu Street	21.82	May-July	48	2.55	3134	<.001
	Dongsha Street	7.56	May-July	33	1.67	477	<.001
**2016**							
	Huangcheng Street	29.03	May-June	41	4.06	6137	<.001
	Shilou Town	21.89	May-June	45	3.56	4817	<.001
**2017**							
	Lutian Town	62.13	August-October	41	2.96	5441	<.001
	Xinzao Town	20.77	June-August	36	2.47	2806	<.001
	Jinsha Street	6.82	June-July	27	2.39	985	<.001
**2018**							
	Liangkou Town	69.47	August-October	39	2.93	3418	<.001
	Shiji Town	20.77	June-August	44	2.27	1721	<.001
	Jinsha Street	6.82	June-August	27	2.2	693	<.001
**2019**							
	Liangkou Town	69.47	May-July	39	3.51	9511	<.001
	Xinzao Town	14.68	May-July	46	2.77	5760	<.001
**2020**							
	Wenquan Town	55.32	September-November	40	5.29	3086	<.001
	Shilou Town	20.98	September-November	42	3.53	1428	<.001
	Sanyuanli Street	8.26	October-November	61	2.35	385	<.001
**2021**							
	Yongning Street	27.44	April-May	48	3.80	5788	<.001
**2022**							
	Xintang Town	28.69	June-August	45	3.79	3446	<.001
	Caihong Street	9.91	June-August	68	2.11	788	<.001

a RR: relative risk.

b LLR: log-likelihood ratio.

### Influencing Factors of HFMD Incidence

Before modeling, correlation analysis was conducted with HFMD incidence as the dependent variable and all potential explanatory variables as independent variables. The collinearity diagnosis results indicated that variables in the same group had multicollinearity problems. The performance of the model was improved by deleting or merging variables with high collinearity, reducing the variance inflation factor of the remaining variables was reduced to less than 10.

Multivariate OLS was used to analyze the relationship between demographic, economic, educational, and medical factors and HFMD incidence (Table 3). Population density was found to be a protective factor for HFMD. In terms of economic factors, average annual income of employees was a protective factor. The impact of medical factors on HFMD incidence was not significant. In terms of educational factors, the average number of students in each primary school and kindergarten were risk factors. The fitting effects of different models are shown in Table 3. The GTWR model had the highest *R*^2^ value and the lowest AIC value, indicating that the GTWR model predicted the relationship between the incidence of HFMD and the above factors better than the OLS and GWR models. In the scatter plot (Multimedia Appendix 4) with the predicted values from the GTWR model on the x-axis and the standardized residuals on the y-axis, the residuals were randomly distributed around 0, with no apparent patterns or trends.

**Figure 6.**
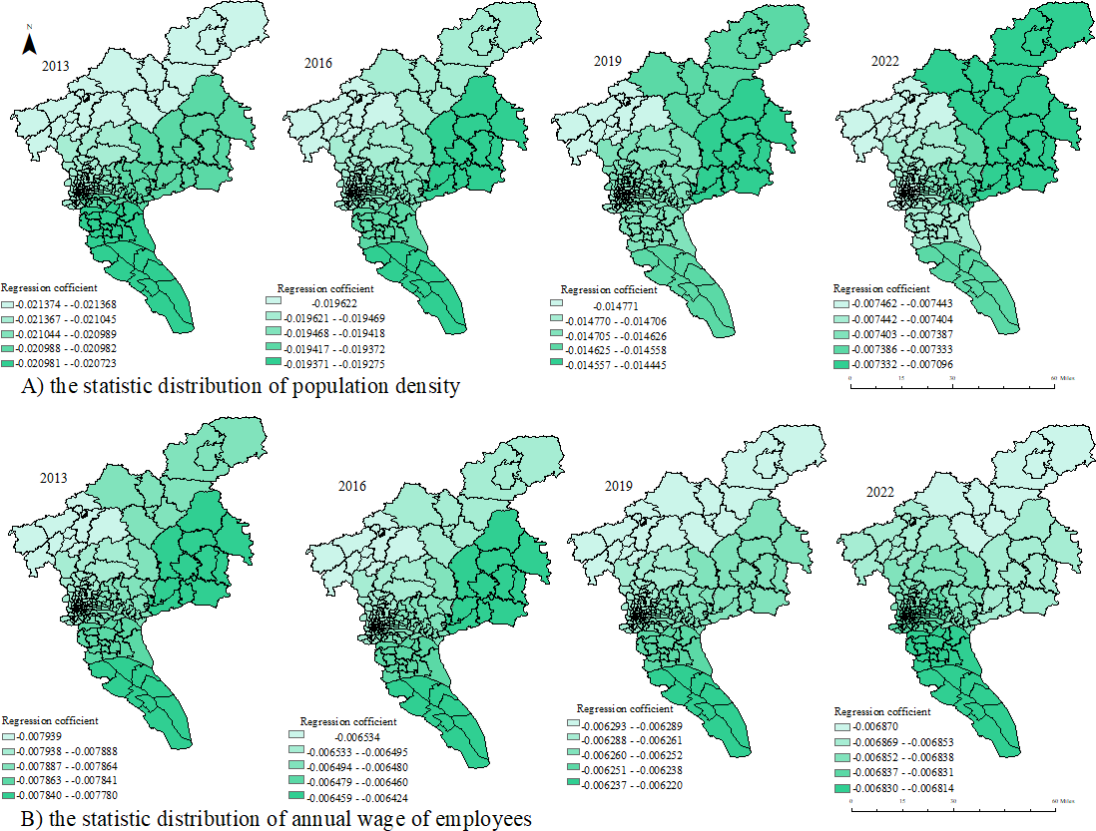
Spatial distribution of regression coefficients of protective factors in the geographically and temporally weighted regression (GTWR) model. A) represents the impact of population density on the incidence of HFMD in different spatial regions over various years. B) represents the impact of annual wage of employees on the incidence of HFMD.

**Figure 7.**
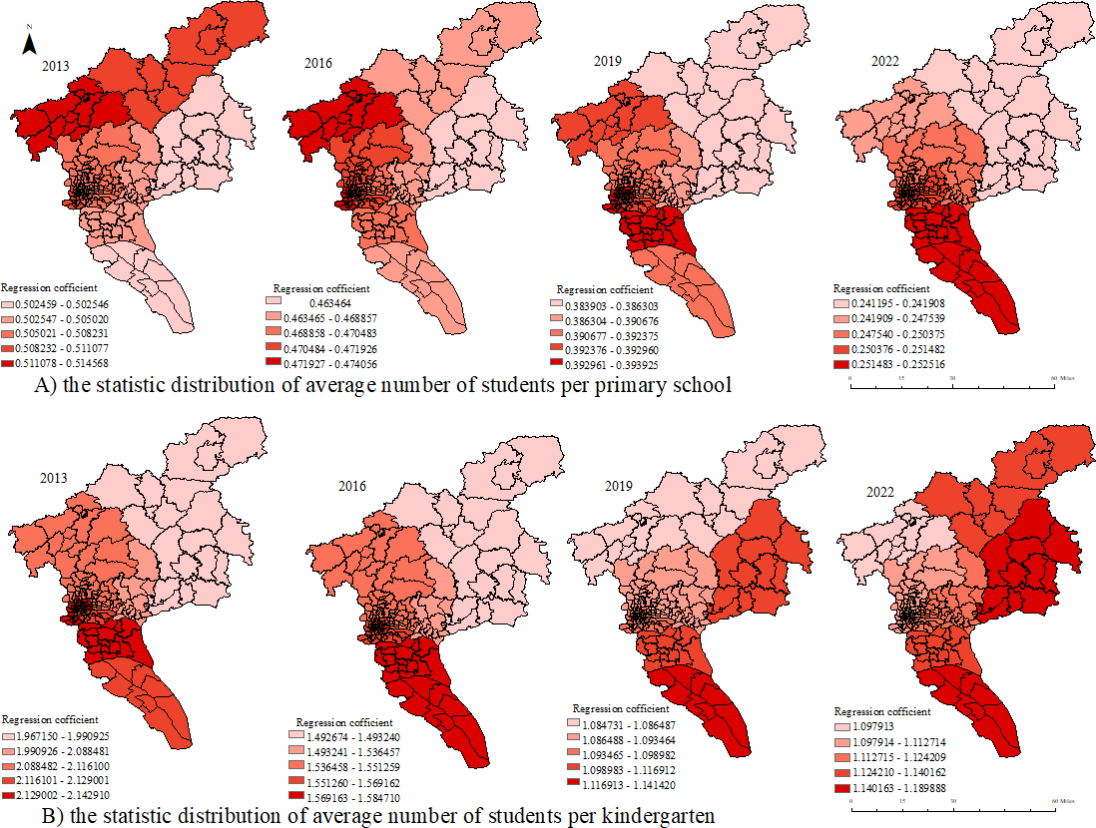
Spatial distribution of regression coefficients of risk factors in the geographically and temporally weighted regression (GTWR) model. A) represents the impact of average number of students per primary school on the incidence of HFMD in different spatial regions over various years. B) represents the impact of average number of students per kindergarten on the incidence of HFMD.

Some results of the GTWR model are shown in Figure 6 and Figure 7. The impact of population density on the incidence of HFMD in the west and north of Guangzhou gradually increases. Additionally, the high-impact area of employee annual wages on HFMD gradually shifts from the northern to the southern regions of Guangzhou. The average number of students per primary school migrates from the west to the south, while the average number of students per kindergarten migrates from the west and south to the east.

**Table 3.** Multivariate ordinary least squares (OLS), geographically weighted regression (GWR), and geographically and temporally weighted regression (GTWR) model results for hand, foot, and mouth disease incidence in Guangzhou.

Variable		Ordinary least regression model		
		β	SE	*P* value
**Population**				
	Population density	−0.017	0.004	<.001^a^
	Married women of reproductive age	1.454	7.033	.83
	Urban population ratio	0.235	1.77	.89
**Economy**				
	Annual wage of employees	−0.066	0.001	<.001^a^
	Per capita gross domestic product	−3.167	2.946	.28
**Education**				
	Average number of students per primary school	0.438	0.092	<.001^a^
	Average number of students per kindergarten	1.478	0.713	.04^b^
**Medical**				
	Health institutions	−0.029	0.16	.86
	Health technicians	0.005	0.004	.22
	Corrected AIC^c^	1447.942	—^d^	—
	Adjusted *R*^2^	0.540	—	—
	Corrected AIC (GWR)	1449.676	—	—
	Adjusted *R*^2^ (GWR)	0.552	—	—
	Corrected AIC (GTWR)	1451.356	—	—
	Adjusted *R*^2^ (GTWR)	0.602	—	—

a *P*<.001.

b *P*<.05.

c AIC: Akaike information criterion.

d Not applicable.

## Discussion

### Principal Results

This study used geospatial analysis methods and GWR models to comprehensively describe the epidemic trend of HFMD in Guangzhou and explore the spatiotemporal distribution patterns and its influencing factors. From 2013 to 2022, a total of 599,353 patients with HFMD were reported in Guangzhou, and children aged 5 years and younger accounted for more than 90% of the total cases. The incidence showed a bimodal distribution, mainly concentrated from May to July, with the subpeak period from August to November, which is consistent with the existing studies in various countries [2,29,30]. There are fewer cases in those aged 10 years and older because almost all HFMD cases in Guangzhou are mild, meaning that even if adults are infected, the symptoms may not be enough to significantly affect their daily lives, resulting in lower reporting rates among adults. The incidence rate fluctuated from 558.40 cases to 403.51 cases per 100,000 people, with an average annual decrease of 12.94%, which is similar to the incidence trend of HFMD in China in recent years [6,31]. However, HFMD is still one of the most serious infectious diseases [32]. During the COVID-19 epidemic, due to lockdown measures and social distancing [33], infants and young children were exposed to fewer viruses and bacteria, resulting in their immune systems not being adequately exercised. Therefore, the prevention and control of HFMD should continue to focus on young children [34], and attention should be paid to home and living environment hygiene, and good hygiene habits should be cultivated in children from an early age.

We conducted spatial autocorrelation analysis and space-time scan statistics analysis of HFMD at street and township scales in Guangzhou, China. The results showed that high-risk areas were mainly distributed in peripheral urban areas (eg, Huadu, Panyu, Conghua, and Zengcheng). The reason may be that the peripheral urban areas are far away from the economic and cultural center of Guangzhou. With the increasing urbanization rate of the central urban areas, the young and middle-aged people in the peripheral urban areas gradually transfered to the central urban areas. The older people and the infants left behind were relatively weak in medical awareness and knowledge of disease prevention. At the same time, the living environment and hygiene habits in the outer urban areas were lower compard to those in central urban areas. In addition, some studies have shown that the change and transformation of industrial structures have an impact on the high-risk areas of HFMD, and the tertiary industry has a greater impact on HFMD than the primary industry [35-37]. Since 2017, Conghua and Zengcheng districts have gradually transformed into commercial districts, industrial parks, tourism, and other service industries [38,39]. The rapid development of the service industry, especially the increase in catering and amusement places, has increased the frequency and scope of children’s gatherings, providing more opportunities for the spread of HFMD. Therefore, the high-risk areas of HFMD were gradually shifted from Huadu District to Conghua and Zengcheng Districts.

Existing studies widely believe that socioeconomic factors are connected to the incidence of HFMD, with population density as a potential determinant in most areas of China [24,40]. A study conducted in China found that the higher population density in developed areas made it easy for the virus to spread [10]. A spatiotemporal analysis of southern China also indicated that HFMD outbreaks occurred earlier as population density gradually increased. Moreover, the areas most prone to clustering were typically the most prosperous [23]. However, this study showed the opposite. The population density in Guangzhou increased by 820 people/km^2^ from 2013 to 2022, and the average annual wage increased from 69,692 to 152,324 Yuan (USD 9,588 to 20,956), while HFMD incidence decreased. Another example is that the population density in the central urban area of Guangzhou exceeds 10,000 people/km^2^, of which Yuexiu District is more than 30,000 people/km^2^, but the incidence rate in the central urban area is lower than that in outer urban areas all year round. This may be due to Guangzhou being different from most regions in China in terms of population, economy, and culture. First, as a very developed city, although the population of Guangzhou is relatively concentrated in a small geographical area, the population structure is dominated by young and middle-aged individuals; specifically, the proportion of people older than 5 years in the central urban area is more than 90%, and the proportion of infants and young children in the key population of HFMD is not high [18]. Second, a cross-sectional survey in Guangzhou [41] showed that just 3 months after the EV71 vaccine was approved, nearly half of parents were willing to pay for their children’s vaccines, which was higher than the willingness and vaccination rate in other cities during the same period [42,43]. A study of the effectiveness of the vaccine conducted by Guangzhou [44] showed that the 2-dose EV71 vaccination was an effective measures to prevent 3-year-old children from being infected with HFMD. A questionnaire survey of 592 parents or legal guardians in 6 kindergartens in Guangzhou showed that the level of hand hygiene and infectious disease awareness of parents or legal guardians was commendable. All these factors show that Guangzhou residents attach great importance to HFMD [45]. In addition, the fecal-oral transmission is one of the routes for intestinal infectious diseases, such as HFMD [2]. Guangzhou’s dietary habits predominantly favor healthy cooking techniques like steaming and stewing. Therefore, the ingredients are of high quality and hygienic standards, and heating them for a long time helps destroy bacteria and viruses in food, thereby reducing the risk of infectious diseases.

We also found that the average number of students per primary school or kindergarten increased the risk of HFMD. Many children in kindergartens congregate in relatively small spaces, and the stagnant indoor air and close contact between children help spread HFMD pathogens. At the same time, inadequate cleaning and disinfection of daily necessities, such as toys, tableware, tables, chairs, and floors, will cause the breeding of pathogens; hand hygiene compliance among children around the world is suboptimal, regardless of whether they are in relatively rich or poor countries [34], which also increases the risk of infection. For example, from 2013 to 2017, the average number of preschool education places in Huadu District continued to grow and ranked in the forefront of Guangzhou, and the number of primary school students also increased from 109,375 to 141,029. Therefore, during this period, the high-risk areas of HFMD in Guangzhou were mainly distributed in Huadu District, while the low-risk areas were distributed in central urban areas, such as Liwan District, where the average number of children per kindergarten was the lowest all year round.

In summary, targeted prevention and control measures can be implemented to further reduce the HFMD incidence. First, while steadily advancing prevention and control measures in the central areas, special attention needs to be paid to prevention and control work in peripheral urban areas. This includes strengthening the management and supervision of places where infants and young children are concentrated, such as kindergartens and schools in peripheral urban areas; building and improving the epidemic monitoring system in peripheral urban areas; and strengthening information sharing and communication among medical institutions, schools, health departments and other units. Furthermore, it is suggested that health authorities implement a free HFMD vaccination program for children younger than 5 years in peripheral urban areas to increase the vaccination rate of infants and young children. Finally, the education department should implement measures to foster a safer environment in preschool education institutions and reduce transmission risks for infectious diseases like HFMD. This includes limiting the number of students in each class to reduce the concentration of infants and young children and carrying out special training on common infectious diseases for teachers in institutions.

### Limitations

This study has some limitations. First, the Guangzhou Statistical Yearbook only collects population data of district-level administrative units, while the population data of streets and towns are estimated based on the data in the 2020 Guangzhou Census Yearbook; therefore, the incidence rate of streets and townships was not accurate enough. Second, the influencing factors of HFMD are complex and diverse. In addition to socioeconomic factors, there are also meteorological and other factors [25,46]. Although there is not much difference in temperature and humidity between districts in Guangzhou, they may also have a certain impact. Third, the symptoms of most HFMD cases are mild and similar to clinical symptoms of other childhood diseases, such as chickenpox [47]. Some parents may not be aware that their infants and young children are sick or may not take them to see a doctor. These cases may affect the results of the regression analysis.

### Conclusions

From 2013 to 2022, there were obvious differences in HFMD incidence among different populations, seasons, and regions in Guangzhou. Children aged 5 years and younger are the high-risk group for HFMD. The hot and humid summer and autumn seasons are the peak periods for the disease. The peripheral urban areas are the main aggregation areas of Guangzhou. Research shows that the average number of students per primary school and kindergarten is the main reason for the increase in the HFMD incidence, while population density and per capita income play a protective role. The results of this study contribute to a deeper understanding of the epidemic characteristics of HFMD in southern China, provide an important basis for formulating targeted prevention and control strategies, and can be a reference for other countries and regions facing similar challenges.

## Supplementary material

10.2196/58821 Multimedia Appendix 1 Description of factors influencing of the hand, foot, and mouth disease (HFMD).

10.2196/58821 Multimedia Appendix 2 The number of hand, foot, and mouth disease (HFMD) cases and incidence rates by age in Guangzhou from 2013 to 2022.

10.2196/58821 Multimedia Appendix 3 The number of hand, foot, and mouth disease (HFMD) cases and incidence rates by district in Guangzhou from 2013 to 2022.

10.2196/58821 Multimedia Appendix 4 Distribution of standardized residuals in the geographically and temporally weighted regression (GTWR) model.

## Abbreviations

AIC: Akaike information criterion
GTWR: geographically and temporally weighted regression
GWR: geographically weighted regression
HFMD: hand, foot, and mouth disease
HH: high-high
LISA: local indicator of spatial association
LLR: log-likelihood ratio
OLS: ordinary least squares
