# Supplementary figures and images for: Spatiotemporal Changes and Influencing Factors of Hand, Foot, and Mouth Disease in Guangzhou, China, From 2013 to 2022: Retrospective Analysis
Source: JMIR Public Health Surveill. 2024 Aug 2;10:e58821. doi: 10.2196/58821 (PMC11310896; doi:10.2196/58821)

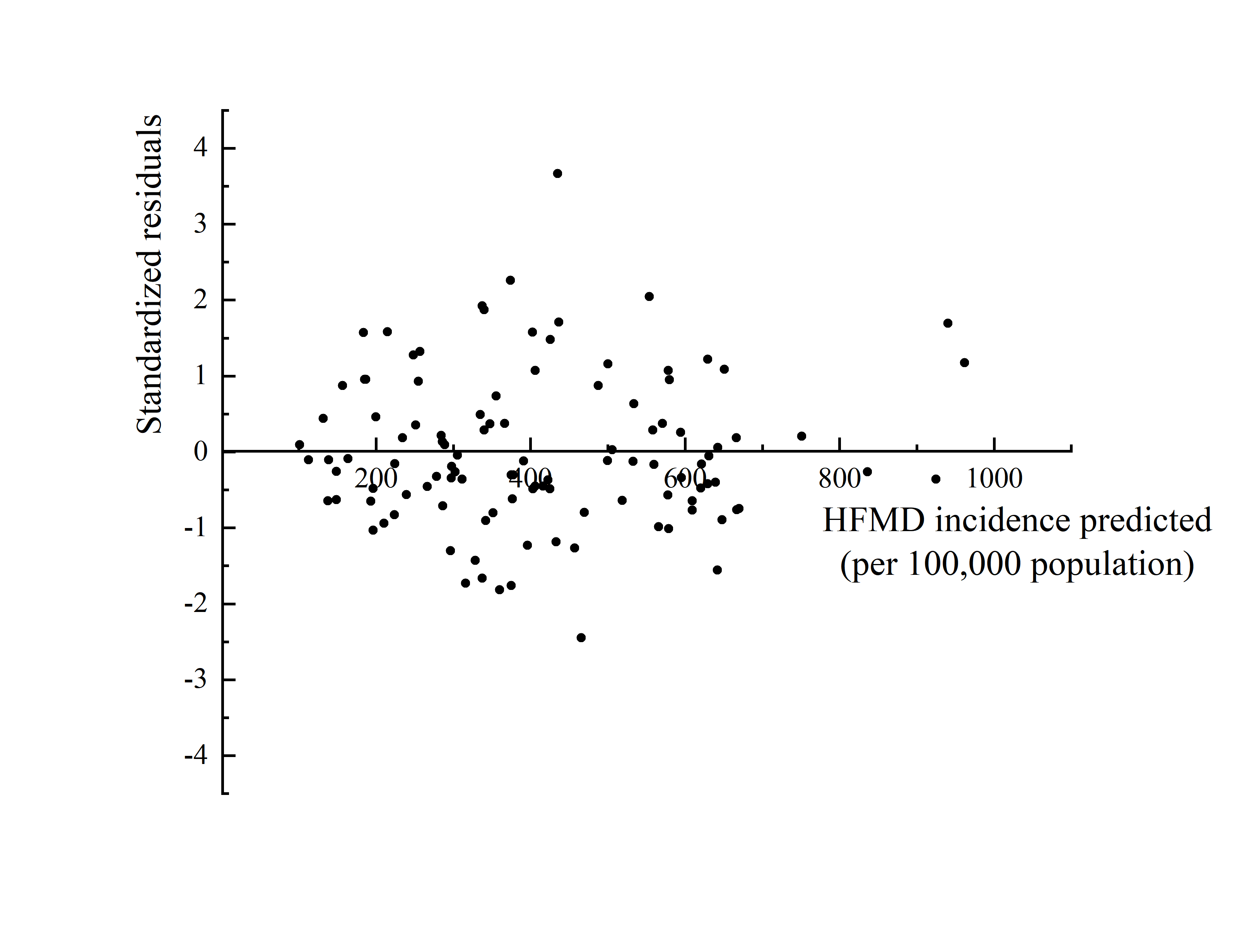

Supplement: Multimedia Appendix 4 [file publichealth-v10-e58821-s004.png]
